# Eye-Safe Lidar System for Pesticide Spray Drift Measurement

**DOI:** 10.3390/s150203650

**Published:** 2015-02-04

**Authors:** Eduard Gregorio, Francesc Rocadenbosch, Ricardo Sanz, Joan R. Rosell-Polo

**Affiliations:** 1 Department of Agricultural and Forest Engineering, Research Group on AgroICT & Precision Agriculture, Universitat de Lleida (UdL), Campus Cappont, Bldg CREA, Pere de Cabrera s/n, 25001 Lleida, Spain; 2 Department of Signal Theory and Communications, Remote Sensing Laboratory, Universitat Polit&cnica de Catalunya (UPC), Campus Nord, Bldg D4, Jordi Girona 1-3, 08034 Barcelona, Spain; E-Mail: roca@tsc.upc.edu; 3 Department of Agricultural and Forest Engineering, Research Group on AgroICT & Precision Agriculture, Universitat de Lleida (UdL), Campus ETSEA, Av/ Rovira Roure 191, 25198 Lleida, Spain; E-Mails: rsanz@eagrof.udl.cat (R.S.); jr.rosell@eagrof.udl.cat (J.R.R.-P.)

**Keywords:** lidar, spray drift, optomechanical design, signal-to-noise ratio, eye safety, pesticide, laser, remote sensing, agriculture

## Abstract

Spray drift is one of the main sources of pesticide contamination. For this reason, an accurate understanding of this phenomenon is necessary in order to limit its effects. Nowadays, spray drift is usually studied by using *in situ* collectors which only allow time-integrated sampling of specific points of the pesticide clouds. Previous research has demonstrated that the light detection and ranging (lidar) technique can be an alternative for spray drift monitoring. This technique enables remote measurement of pesticide clouds with high temporal and distance resolution. Despite these advantages, the fact that no lidar instrument suitable for such an application is presently available has appreciably limited its practical use. This work presents the first eye-safe lidar system specifically designed for the monitoring of pesticide clouds. Parameter design of this system is carried out via signal-to-noise ratio simulations. The instrument is based on a 3-mJ pulse-energy erbium-doped glass laser, an 80-mm diameter telescope, an APD optoelectronic receiver and optomechanically adjustable components. In first test measurements, the lidar system has been able to measure a topographic target located over 2 km away. The instrument has also been used in spray drift studies, demonstrating its capability to monitor the temporal and distance evolution of several pesticide clouds emitted by air-assisted sprayers at distances between 50 and 100 m.

## 1. Introduction

The application of plant protection products by means of sprayers is the most widely used procedure for the protection of agricultural crops against pests and diseases. Spray drift is defined by the standard ISO 22866:2005 as the quantity of plant protection product that is carried out of the sprayed (treated) area by the action of the air currents during the application process. Most airborne spray drift measurements carried out today are made using collectors and tracers. The use of this type of methodology is costly and time-consuming. Furthermore, the information on the pesticide cloud is not time resolved, volume imaging of the cloud is not possible and collector efficiency is influenced by meteorological conditions [1]. In addition, because of the extensive variety of crop and meteorological conditions it is difficult to make an accurate assessment of the real spray drift hazard associated with each application technique. As a result, there has been a growing interest in the search for alternative methods which can be used either in the laboratory, with wind tunnels, or in the field. The use of optical systems like lidar (light detection and ranging) has emerged as one of the most feasible options.

In the last 25 years lidar systems have been applied in several spray drift studies, some of the most relevant are cited below. The Atmospheric Environment Service of Canada constructed a Nd:YAG elastic-backscatter lidar (1064 nm, 50 mJ) to obtain near-real-time maps of the pesticide plumes in aerial applications [2–4]. This instrument allows to study the dynamics of the pesticides and the influence over them of aircraft wing-tip vortices. A more powerful Nd:YAG lidar system (1064 nm, 125 mJ), developed by researchers from the University of Connecticut, has been used to validate theoretical spray-drift models [5], to assess the influence of atmospheric stability over spray drift movement [6], to generate tri-dimensional images of the spray drift plume over an orange orchard [7] and to estimate the concentration of the pesticide cloud from the backscatter lidar signal [8,9]. In [10], researchers from the University of Washington at Seattle monitored the pesticide plume over an apple orchard using an UV lidar (355 nm, 8 mJ) and compare these measurements with a spray simulation model. In a recent study [1], the authors of the present paper have obtained a strong relationship between spray drift measurements using simultaneously an UV lidar (355 nm, 16 mJ) and passive collectors.

These studies show that lidar systems allow real-time monitoring of airborne spray drift, obtaining range-resolved images of the spray plume while requiring fewer personnel and consuming less time. However, despite the advantages of lidar systems for airborne spray drift monitoring, they have been used in only a limited way to date. This is because currently available lidar systems inherited their design architecture from atmospheric monitoring applications (high energy, low pulse-repetition-frequency systems), which make them expensive and require trained personnel for their operation [11]. In addition, many of these instruments are not eye-safe, preventing their practical application particularly in ground spray drift studies.

In order to overcome previous limitations, this work presents the development of a new lidar system specifically suited for pesticide spray drift monitoring. Design specifications which this instrument needs to satisfy are:

▪ Compactness. An easily transportable instrument suitable for field work is required.
▪ Near-field and distance resolution measurement capabilities. Drift plumes generated by ground sprayers have relatively low dimensions, commonly just a few metres thick. For appropriate characterisation, the lidar system must have a high range resolution, ideally not greater than 3 m. A maximum reach of 500 m is sufficient.
▪ Temporal resolution capability. Pesticide plumes are highly dynamic, with rapid variations in their shape and concentration. In order to characterise these clouds, the lidar system must be capable of measurements at high frequencies. It was experimentally shown in [1] that a time resolution of 1 s is suitable for the monitoring of spray drift plumes.
▪ Eye safety. The drift clouds generated by ground-based applications are usually suspended at a low height above the sprayed crop [7]. Therefore, monitoring pesticide drift with terrestrial lidar systems implies a quasi-horizontal sounding, increasing the risk of accidental impinging on bystanders. It is therefore concluded that the instrument must be eye-safe (IEC/EN 60825).

This paper is organised into five sections. Section 1 comprises this introduction. In Section 2, the principal design parameters are established via signal-to-noise ratio simulations and attending eye safety requirements. Section 3 shows the different components that comprise the emitter and receiver subsystems. Initial experimental measurements with the constructed prototype are presented in Section 4. Finally, Section 5 gives the concluding remarks.

## 2. Performance Assessment

In this section, the wavelength, pulse energy and receiving area are determined by taking into account the design specifications of Section 1. First, Section 2.1 studies the eye safety corresponding to several wavelengths typically used in lidar systems. As a result of this study, a shortlist of three possible wavelengths is obtained: 905 nm, 1064 nm and 1.5 μm. In Section 2.2, it is established for each of these wavelengths the pulse energy and receiving area intervals required to detect a drift cloud located at 500 m. This analysis is carried out by signal-to-noise (SNR) simulation considering the atmospheric model defined in Section 2.2.1 and the SNR expression presented in Section 2.2.2. Available photodetectors in each of the wavelengths are assumed in the simulations. In Section 2.3, based on the pulse energy previously obtained, the required laser beam expansion for each wavelength is calculated to reach eye safety. A wavelength of 1.5 μm is selected since the beam expansion is moderate, satisfying the design specification of compactness established in Section 1.

### 2.1. Maximum Permissible Exposure for Different Wavelengths

Wavelength is one of the key parameters in the design of any lidar system. Dependant on this parameter are the laser emitters and photodetectors that can be used, the mechanisms of interaction with the atmosphere and the eye safety level that will be required. As the design starting-point, a comparative analysis is conducted in this section of the maximum permissible exposure (MPE) to the following wavelengths:

▪ λ = 355 nm. Typical of UV lidars [12], corresponding to the third harmonic of the Nd:YAG solid state laser.
▪ λ = 523 nm. Visible radiation used by the Micro Pulse Lidar [13].
▪ λ = 905 nm. Commonly applied in lidar ceilometry [14], corresponding to the InGaAs laser diode.
▪ λ = 1064 nm. IR radiation [15] generated by the Nd:YAG laser (fundamental frequency).
▪ λ = 1.5 μm. Commonly used in eye-safe systems [16].

**Figure 1.**
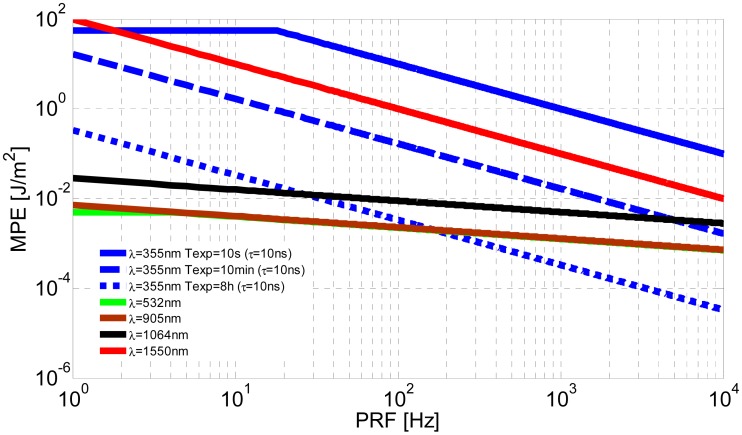
MPE for an individual pulse *vs.* pulse repetition frequency (PRF).

Figure 1 shows the maximum permissible exposure for a single pulse as a function of the pulse repetition frequency. This graph was generated considering for each wavelength and emission frequency the most restrictive of the criteria defined by IEC/EN 60825.

It can be seen that at 355 nm the safety level varies substantially with the exposure time. So, for exposures of 10 s this is the safest wavelength, whereas for more prolonged exposures (and high PRFs) it is one of the most dangerous. This behaviour is due to the UV-A radiation doses being additive. At 532 and 905 nm the MPEs are very similar, while for 1064 nm the safety threshold is approximately twice as high. The 1.5 μm wavelength is known to be eye-safe, though this condition depends on the emitted radiant exposure. It can be seen that at 1.5 μm, permissible exposure is appreciably higher than in the visible or near infrared. In addition, unlike in the ultraviolet, there are no additive photochemical effects in the IR-B.

### 2.2. Signal-to-Noise Ratio Simulations

In this section, the interval of values is determined in which the instrument system constant *K_s_* must be found to satisfy the initial specification of pesticide cloud measurement at a distance of 500 m. For this purpose, SNR simulations were conducted for three wavelengths: 905 nm, 1064 nm and 1.5 μm. Emission at 355 nm was not considered since the doses are additive in the UV-A region and consequently the safety level falls drastically for prolonged exposures. Another drawback of UV-A radiation is that it requires special optical material, since optical glass is not transparent and molecular backscatter in the ultraviolet range is very high [17]. Emission at 523 nm was also disregarded as it has MPE values similar to at 905 nm, but at this latter wavelength solar radiation (background noise) and atmospheric extinction are lower.

#### 2.2.1. Atmospheric Model

As explained previously, the lidar system sounding of the atmosphere will be horizontal and so a homogenous optical atmospheric model is considered in the SNR simulations. Clear-air oversimplified atmospheric conditions are assumed in this model (15 km visibility at 550 nm) and the total extinction and backscatter coefficients, α*_tot_* and β*_tot_*, respectively, are obtained as the sum of the opto-atmospheric components due to aerosols (α*^aer^*, β*^aer^*) and molecules (α*^mol^*, β*^mol^*). The calculation of each of these components is given below.

The molecular (Rayleigh) extinction coefficients at different wavelengths are obtained by applying the following expression:

(1) $$\alpha^{\text{mol}}\left(\lambda \right)={\left(\frac{550}{\lambda \left[nm\right]}\right)}^{4}\alpha_{550}^{\text{mol}}$$

where $\alpha_{550}^{\text{mol}}=0.0116\;{\text{km}}^{-1}$ is the molecular extinction at 550 nm [18].

The molecular backscatter coefficient is a constant multiple of the coefficient of extinction and is given by the relationship:

(2) $$\beta^{\text{mol}}\left(\lambda \right)=\frac{3}{8\pi }\alpha^{\text{mol}}\left(\lambda \right)$$

With known atmospheric visibility *V_M_* [km], the particulate (Mie) extinction coefficient $\alpha_{550}^{\text{aer}}\;[{\text{km}}^{-1}]$ at 550 nm is calculated by applying Koschmieder’s relationship [19]:

(3) $$\alpha_{550}^{\text{aer}}=\frac{3.91}{V_{M}\;[km]}$$

At different wavelengths, extinction due to aerosols is given by:

(4) $$\alpha^{\text{aer}}=\alpha_{550}^{\text{aer}}\;{\left(\frac{550}{\lambda \left[nm\right]}\right)}^{1.3}$$

For link-budget studies, as is the case here, a typical Mie backscatter coefficient $\beta_{550}^{\text{aer}}\;[{\text{km}}^{-1}\cdot {\text{sr}}^{-1}]$ at 550 nm is well assumed for different atmospheric visibility conditions [18]. At other wavelengths, the backscatter coefficient is calculated by applying:

(5) $$\beta^{\text{aer}}=\beta_{550}^{\text{aer}}\;\frac{550}{\lambda \left[nm\right]}$$

Table 1 gives, for each of the wavelengths under consideration, the total coefficients of atmospheric extinction α*_tot_* and backscatter β*_tot_*, calculated through Equations (1)–(5). The values of the diffuse component of solar radiation *L_b_* [20] are also shown.

In the SNR simulations to be presented next, the presence of a pesticide cloud located at 500 m is considered. Extinction or backscatter coefficient values for spray drift clouds have not been found in the literature. For this reason, standard values for low-water clouds (α*_cloud_* = 10 km^−1^, β*_cloud_* = 0.5 km^−1^·sr^−1^, [18]) were used in this study, this being a conservative approximation.

#### 2.2.2. Signal-to-Noise Ratio Equation

A study is undertaken in the simulations of how the SNR varies with the system constant *K_s_* [W·m^3^]. The aim is to find the values of the system constant, *K_s_*, which allow SNR values higher than 5 to be reached. This threshold is considered sufficient for the application of most common automatic detection algorithms [21]. Since the lidar system should measure at high temporal resolution, the required SNR values must be computed for single pulse emission. The SNR expression for single pulse emission is given by [14]:

(6) $$\text{SNR}(R)=\frac{\xi_{0}K_{s}U(R)}{{\left[\frac{2qF}{R_{io}}\left(K_{s}U(R)+K_{b}L_{b}\right)\xi_{0}+{\text{NEP}}_{m}^{2}\right]}^{1/2}B_{N}^{1/2}}$$

*K_s_* [W·m^−3^] is the system constant given by:

(7) $$K_{s}=\frac{E_{0}A_{r}c}{2}$$

where *E*_0_ [J] is the energy emitted per laser pulse, *A_r_* [m^2^] is the effective receiver area and *c* [ms^−1^] the speed of light.

*K_b_* [m^2^·nm·sr] is the background-radiance system constant, given by:

(8) $$K_{b}=A_{r}\Omega_{r}\Delta \lambda$$

where Ω*_r_* [sr] is the receiver-system acceptance solid angle and Δλ [nm] is the interference filter bandwidth.

*U*(*R*) [m^−3^] is the lidar backscattered signal from range *R* [m], given by:

(9) $$U(R)=\frac{\beta_{\text{tot}}(R)}{R^{2}}\exp\left[-2\int_{0}^{R}\alpha_{\text{tot}}(r)dr\right]$$

where β*_tot_*(*R*) [m^−1^·sr^−1^] is the total atmospheric volume backscattering coefficient, *R* [m] is the range and α*_tot_*(*r*) [m^−1^] is the total atmospheric volume extinction coefficient.

ξ_0_ is the optics transmission factor, *R_io_* [A/W] is the photodetector intrinsic current responsivity, *q* [C] is the electron charge, *F* is the excess noise factor, ${\text{NEP}}_{m}\;[\text{W}/\sqrt{\text{Hz}}]$ is the noise equivalent power of the photoreceiver module and *B_N_* [Hz] is the equivalent noise bandwidth at reception.

#### 2.2.3. Signal-to-Noise Ratio Simulations at 905 nm

In the SNR simulations which are presented below, various photodetector types were considered and the value of the optics transmission factor ξ_0_ was tuned as shown in the variants of Table 2.

At wavelengths of 905 and 1064 nm there are three photodetector options: silicon PIN photodiodes, silicon avalanche photodiodes (APD) and photomultiplier tubes (PMT). In this design it was decided to use photodiodes given their superior quantum efficiency in comparison with PMTs.

A PIN photodetector module and an APD module were considered in the simulations that were performed. Both modules are comprised of a photodiode and transimpedance amplifier (TIA), with the latter being the element which limits the noise-equivalent bandwidth *B_N_* of the receiver.

Noise-equivalent bandwidth. In order to determine *B_N_*, a range resolution of Δ*R* = 1 m is considered in these simulations. When applying Δ*R* = *c*(τ*_l_* + τ*_d_*)/2 [18], a bandwidth greater than 107 MHz is required. In this calculation, a pulse duration τ*_l_* ≤ 2 ns and a detection time given by τ*_d_* = 1/2*B_N_* [22] are assumed. In a photodiode-based (APD/PIN) optoelectronic receiving chain, *B_N_* is the transimpedance amplifier (TIA) bandwidth because its bandwidth is much smaller than that of the photodiode (typically, a few GHz). At the receiver output, the signal must be sampled at a frequency *f_s_* ≥ 2*B_N_* according to Nyquist’s criterion (that is, *f_s_* ≥ 215 MHz considering the example figures given). It is assumed that both receiver modules are comprised of a TIA with a bandwidth of 200 MHz and a typical input noise current $I_{a}=6.5\;\text{pA}/\sqrt{\text{Hz}}$.

APD module noise-equivalent power. A noise equivalent power *NEP_m_* of $0.105\;\text{pW}/\sqrt{\text{Hz}}$ is assumed for the APD module. This value is obtained from the equation [23]:

(10) $${\text{NEP}}_{m}=\frac{I_{\text{noise}(\text{total})}}{M\cdot R_{io}}$$

where *I*_*noise*(*total*)_ [ $\text{pA}/\sqrt{\text{Hz}}$ ] is the total input noise-current spectral density. The transimpedance amplifier nearly always generates much more noise than the photosensitive diode and, therefore, the approximation *I*_*noise*(*total*)_ ≈ *I_a_* has been used. For the APD receiver module, intrinsic responsivity *R_io_* = 0.62 A/W at 905 nm and a gain *M* = 100 are considered. An excess noise factor *F* = 4 is estimated, with application of the empirical formula *F* = *M^x^* [24], where *x* = 0.3 is the excess noise index for a Si-APD.

PIN module noise-equivalent power. Intrinsic responsivity *R_io_* = 0.62 A/W at 905 nm is also considered for the PIN module. In this case, there is no gain (*M* = 1) and so the excess noise factor is *F* = 1. The noise equivalent power is $10.49\;\text{pW}/\sqrt{\text{Hz}}$, a value calculated through Equation (10).

Optical transmissivity. Three values for the transmission factor were simulated, corresponding to high (ξ_0_ = 0.5), moderate (ξ_0_ = 0.25) and low (ξ_0_ = 0.1) optical transmissivity.

Background-radiance system constant. For *K_b_* a value of 6.17 × 10^−9^ m^2^·nm·sr is assumed. This parameter was calculated by applying Equation (8), taking a reception diameter of 50 mm, a field of view of 1 mrad and an interference filter width of 1 nm.

**Figure 2.**
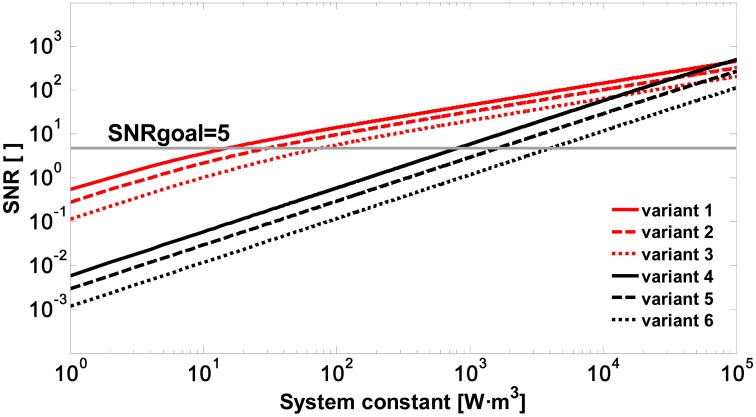
Signal-to-noise ratio *vs.* system constant due to a spray drift cloud located at 500 m for variants 1 to 6 (Table 2).

In Figure 2, the signal-to-noise ratio is simulated as a function of the system constant *K_s_* (variants 1 to 6 in Table 2) in response to the lidar backscattered signal of Equation (9) and the optical coefficients of Table 1. It can be seen that when using an APD module (in red, Figure 2), it is possible to achieve an SNR of 5 with system constants (*K_s_* between 16.33 and 81.58 W·m^3^ in Table 2) two orders of magnitude lower than those required when using a PIN module (*K_s_* between 852.3 and 4261 W·m^3^ in Table 2). Therefore, using a Si-APD module and assuming reception diameters *D_r_* of between 50 and 100 mm, it is concluded that the required pulse energy must be between 14 and 277 μJ (Equation (7)).

#### 2.2.4. Signal-to-Noise Ratio Simulations at 1064 nm

The same photodetector modules, transmission factors and background-radiance system constant as for the 905 nm simulations were considered in the simulations at 1064 nm. It should be noted that at 1064 nm, the intrinsic responsivity *R_io_* of the photodiodes is 0.34 A/W. Applying Equation (10), a *NEP_m_* of 0.19 and $19.12\;\text{pW}/\sqrt{\text{Hz}}$ is obtained for the APD and PIN modules, respectively.

**Figure 3.**
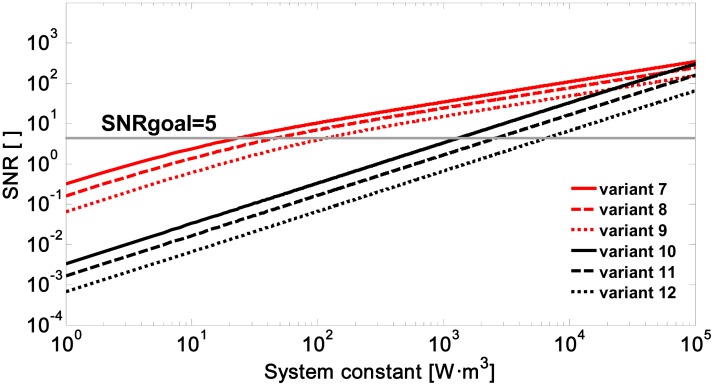
Signal-to-noise ratio *vs.* system constant due to a spray drift cloud located at 500 m for variants 7 to 12 (Table 3).

Figure 3 shows the signal-to-noise ratio *vs.* the system constant for variants 7 to 12. As at 905 nm, it can be seen that using APD modules system constants are required (*K_s_* between 28.95 and 144.7 W·m^3^ in Table 3) some two orders of magnitude lower than for a PIN module (*K_s_* between 1513 and 7568 W·m^3^).

It is concluded that at a wavelength of 1064 nm a system constant is required to be between 1.2 and 1.8 times higher than for 905 nm. This is due to the fall of the intrinsic responsivity of silicon photodiodes at this wavelength, which is due to a reduction in quantum efficiency from 85% to 40%. Using an APD module entails pulse energies (assuming *D_r_* = 50–100 mm) of between 25 and 492 μJ.

#### 2.2.5. Signal-to-Noise Ratio Simulations at 1.5 μm

InGaAs or Ge photodiodes are commonly used at 1.5 μm. Though InGaAs photodiodes are more expensive, they have higher bandwidth and less noise than the Germanium type. In this section, an APD photodetector module and a PIN module are simulated, both based on InGaAs diodes.

Standard values of intrinsic responsivity *R_io_* = 0.93 A/W, gain *M* = 10 and excess noise factor *F* = 5.5 are assumed for the APD module. The same intrinsic responsivity is assumed for the PIN module, but in this case there is no gain (*M* = 1) and so *F* = 1. It is assumed that these photodetector modules incorporate a TIA with the same noise and bandwidth characteristics as those considered in the simulations at 905 nm (*B_N_* = 200 MHz, $I_{a}=6.5\;\text{pA}/\sqrt{\text{Hz}}$). Applying Equation (10), a *NEP_m_* of 0.7 and $7\;\text{pW}/\sqrt{\text{Hz}}$ is obtained for the APD and PIN modules, respectively. Three optical transmissivity values are also considered: ξ_0_ = 0.5, 0.25 and 0.1.

**Figure 4.**
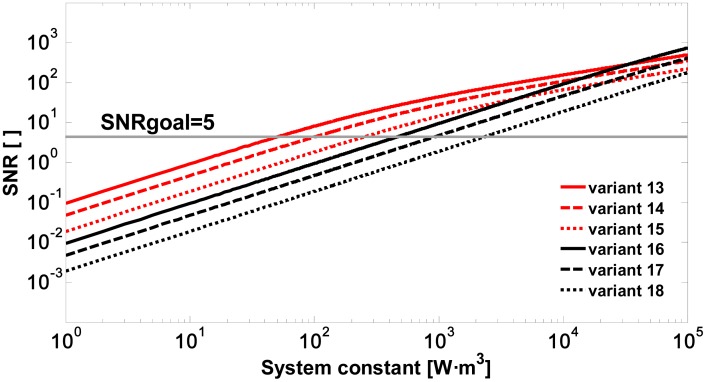
Signal-to-noise ratio *vs.* system constant due to a spray drift cloud located at 500 m for variants 13 to 18 (Table 4).

It can be seen in the simulations of Figure 4 that with an APD module an SNR of 5 is achieved for values of *K_s_* (between 58.29 and 291.5 W·m^3^ in Table 4) one order of magnitude lower than when using a PIN module (*K_s_* between 530.7 and 2654 W·m^3^ in Table 4).

For a wavelength of 1.5 μm and using InGaAs APD modules, system constants between 2 and 3.6 times higher are required when compared with the simulations at 905 nm. Assuming values of *D_r_* = 50–100 mm, it is concluded that the required pulse energies range between 50 and 1000 μJ.

### 2.3. Selection of the Wavelength

Table 5 shows for each studied wavelength at several pulse repetition frequencies (PRF) the laser beam expansion required for the system to be compliant with eye-safe regulation IEC/EN 60825 (see Section 2.1). The starting point for this involves the MPE levels presented in Section 2.1 and the pulse energy intervals calculated in Section 2.2. The PRF values listed consider that the system must have a temporal resolution higher than 1 s, so a minimum PRF of 1 Hz is required. The higher the PRF, the higher the temporal resolution, though the eye safety level will be lowered.

At 905 nm eye safety is only achieved for beam expansions greater than 50 mm (Table 5). At 1064 nm the MPE values are reached with beam expansions slightly lower than those required at 905 nm. This is because of the higher eye safety level at this wavelength and despite the emission of higher pulse energies. At 1064 nm, beam diameters greater than 50 mm are required in all cases, except for low energy emissions and moderate PRF values (25 μJ and 1–10 Hz, Table 5). The beam expansions required at 1.5 μm are between one and two orders of magnitude lower than those calculated for 905 and 1064 nm.

Based on the above results, the option chosen was 1.5 μm. This allows emission of the required pulse energies, while at the same time meeting the compact design requirements specified initially. Apart from the advantages in terms of eye safety, at this wavelength background solar radiation is approximately one order of magnitude lower than at 1064 nm and the Rayleigh signal is small. One drawback that should be mentioned is that the InGaAs APDs available at 1.5 μm have maximum diameters of just 200 μm. These small sizes limit the field of view and introduce greater demands on the optomechanical design. Despite this, 1.5 μm wavelength emission constitutes, at the present time, one of the most promising alternatives for the development of eye-safe lidar systems [17].

## 3. Optomechanical Design

**Figure 5.**
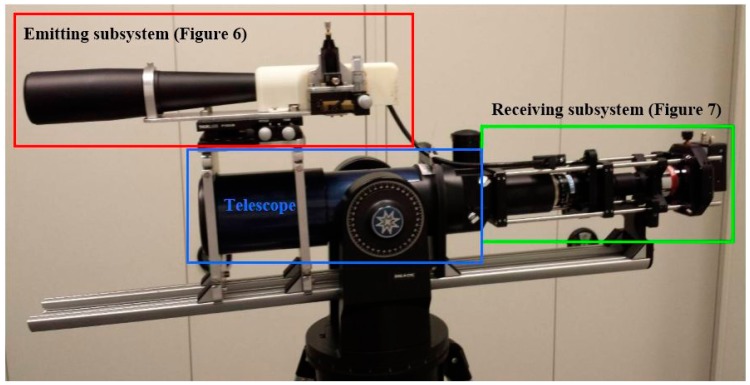
Picture of the lidar prototype showing the emitting and receiving subsystems.

This section presents the optomechanical configuration of the constructed prototype. The different components are chosen on the basis of the parameters established in Section 2. It was decided to opt for a biaxial configuration as, unlike a coaxial configuration, it does not require compensation systems for the internal optical cross talk. Figure 5 shows the optomechanical configuration of the constructed prototype and Table 6 presents the system specifications.

### 3.1. Emitting Subsystem

There are various options for generating pulsed laser energy at 1.5 μm: stimulated Raman scattering (SRS), optical parametric oscillators (OPO), erbium-doped glass lasers and InGaAsP laser diodes. While semiconductor diodes represent the simplest and most economical solution, their low power restricts their application to lidar ceilometers whose energy requirements are not very demanding (∼1 μJ). Other drawbacks of laser diodes include their high divergence and low spectral purity.

Raman scattering has been used in several lidar systems to generate 1.5 μm radiation [16,25–28]. This method consists of passing Nd-YAG radiation through a cell containing methane or deuterium at high pressure to shift the 1.06 μm Nd-YAG output to 1.54 μm. Using SRS, lidar pulse energies up to 225 mJ at 10 Hz of repetition rate have been achieved by [29]. One of the drawbacks of such systems involves safety problems associated with the handling of high pressure cells.

Optical parametric oscillators (OPO) are based on the emission of a laser beam that is directed into a nonlinear crystal placed inside a resonant cavity. This interaction allows the conversion of light from a shorter to longer wavelength [30]. OPOs have been used by several authors as emission sources in eye-safe lidar systems [31,32]. In contrast with SRS techniques, OPO is a solid-state method which allows more compact designs and requires no handling of high-pressure cells. The high cost is its main disadvantage.

Erbium-doped glass lasers in the form of rods or optical fibres directly emit pulses at a wavelength of 1.5 μm. It was decided in this design to opt for a source of this type with its major advantages of simplicity and cost in comparison with the OPOs. Their pulse energies vary from a few microjoules up to 40 mJ [33]. These values are lower than those obtained with SRS or OPOs but they are sufficient for our application. Other examples of lidar systems based on erbium-doped glass lasers can be found in [34,35].

A Kigre™ MK-85 erbium-doped glass laser model (Kigre, Inc., Hilton Head, SC, USA) with a pulse energy of 3 mJ at 1534 nm was used as emission source. This model allows the combination of short pulse durations (<6 ns, Table 6) with low repetition frequencies (limited for eye safety reasons). The chosen unit allows adjustment of the PRF from single shot emissions to 10 Hz. As the pulse energy is greater than the 1000 μJ specified in Section 2.2.5, this allows to compensate for any uncertainties included in the signal-to-noise ratio simulations.

The laser beam has a diameter of 0.8 mm and a divergence of 4.2 mrad. A beam expander with a power of 20× is used to ensure eye safety levels. At the expander output, the laser beam has a diameter of 16 mm, a divergence of around 210 μrad and is fully eye-safe (class 1M, IEC/EN 60825).

**Figure 6.**
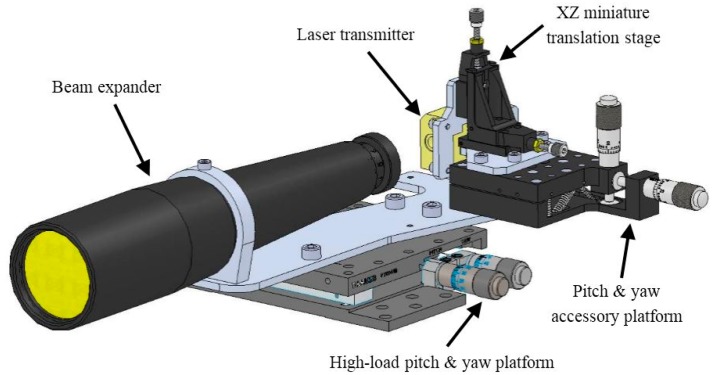
CAD 3D model of the optomechanical emission subsystem.

Figure 6 shows the relative position of the laser transmitter and the beam expander. The laser unit is held in place by means of an XZ miniature translation stage whose function is to adjust the position of the emitted beam with the optical input of the beam expander. This translation stage is in turn held in place by means of a pitch and yaw accessory platform that allows angular alignment of the laser beam with respect to the optical axis of the expander. The whole emission subsystem (laser transmitter and beam expander) is held in place by a high-load pitch & yaw platform that allows precise adjustment of the tilt angle between the optical emission and reception axes. The overlap factor (OVF) and the range at which full OVF is achieved are critically dependent on the tilt angle. More information about the parameters which condition the OVF can be found in [20].

### 3.2. Receiving Subsystem

**Figure 7.**
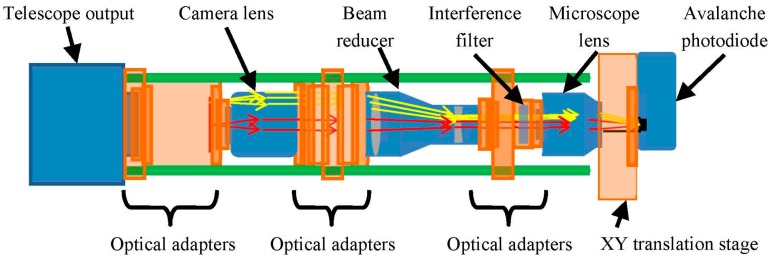
Lidar light collection optics from the rear of the telescope to the APD module.

The optoelectronic receiver is an APD110C model (Thorlabs, Newton, NY, USA) which includes an InGaAsP APD photodiode with an intrinsic responsivity of 0.9 A/W (*M* = 10) and a transimpedance amplifier with a gain of 10^5^ V/A. The photosensitive surface of the APD has a diameter of just 200 μm and, therefore, its correct positioning requires the use of a precision XY translation stage (Figure 7). The main characteristics of the receiver module are shown in Table 6.

An outline of the receiver optics of the lidar prototype is shown in Figure 7. The backscattered energy is captured by a Meade™ ETX 80 reflector telescope (Meade Instruments Corp. Irvine, CA, USA) (Figure 5) with an aperture of 80 mm and focal length equal to 400 mm. Three optical elements are used to focus the light collected by the telescope onto the photodiode: a camera lens, a beam reducer and a reflective microscope objective. The camera lens is reversed so that the focal plane corresponds to the real image created at the output of the telescope. This means that light from the real image is collimated into a low-divergence beam. The beam reducer (×3) diminishes the beam with a factor of three. This ensures that all of the collected light can enter the aperture of the microscope lens. The reflective microscope objective (×15) focuses the light on the photodiode. An interference filter matching the wavelength of the laser source is inserted between the beam reducer and the microscope objective. This is used to remove the ambient light not backscattered by the target, and to increase the signal-to-noise ratio. At this position, the collimated light provided by the beam reducer incises orthogonally on the interference filter surface, avoiding its detuning [23].

The analogue signal from the photodetector module is digitalized using a Gage™ CompuScope 12502 analogue-digital converter (ADC) (DynamicSignals LLC. Lockport, IL, USA) and transmitted to the processing unit (PC). The selected digitizer has 2 channels with a sampling rate of 500 MS/s and 12 bits of vertical resolution. The system has a range resolution of 2.4 m, similar to other 1.5 μm lidar systems [25]. This high resolution is appropriate for the measurement of thin spray drift clouds.

## 4. Experimental Measurements

The first experimental measurements made with the constructed prototype are presented in this section. In an initial stage (Section 4.1), the lidar was used to measure various types of topographic targets (solid objects) for the purpose of adjusting the degrees of freedom of the system. In the second stage (Section 4.2) spray clouds (water) were measured to demonstrate the capacity of the instrument to monitor drift.

### 4.1. Measurement of Topographic Targets

**Figure 8.**
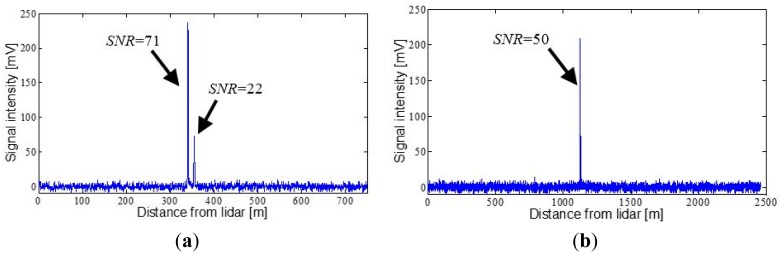
Range profile of lidar signal backscattered by several topographic targets. (**a**) Peaks corresponding to the crowns of a group of trees; (**b**) Detection of a building located at 1125 m from the lidar system.

**Figure 9.**
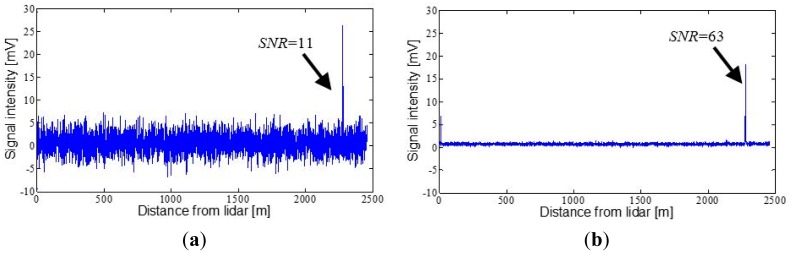
Range profile of lidar signal backscattered by a mountain located at 2275 m from the lidar. (**a**) Return corresponding to a single laser shot; (**b**) Return after averaging 100 laser pulses.

The measurements shown in Figures 8 and 9 were taken on 18 July 2014, after positioning the lidar system on the flat roof of the D4 building of the School of Agrifood and Forestry Science and Engineering (Spanish initials: ETSEA) of Universitat de Lleida, in Lleida (Catalonia, Spain) and aiming it at various surrounding topographic targets. Figure 8a corresponds to the signal backscattered by the crowns of a group of trees located 340 m from the lidar system. Two signal peaks separated by 15 m can be observed. This is because the crowns are comprised of a set of non-homogeneous elements which favour partial impact of the beam on them. In Figure 8b the measurement is shown of the wall of a building located 1125 m away. The SNR estimated for measurement of the trees is 71 (highest peak), while the corresponding SNR for the signal backscattered by the wall is 50. The procedure explained in [14] was used to calculate the SNR.

Figure 9a shows the signal peak backscattered by a mountain located at a distance of 2275 m. The estimated SNR is 11, a far higher value than the threshold of 5 considered in the simulations of Section 2. This result shows that the system is capable of detecting with clarity topographic targets over 2 km away. The measurement shown in Figure 9b corresponds to the same mountain, but in this case averaging a total of 100 shots, (PRF = 2 Hz, observation time of 50 s) in order to reduce the white noise. It can be observed how the SNR improves up to a value of 63. Due to their high dynamics, shot averaging cannot be used for the measurement of drift clouds in cases in which there is a desire to know their temporal evolution. However, this method raises the possibility of monitoring other cloud typologies with slower dynamics, as for example of atmospheric clouds (ceilometry) or those generated in forest fires.

### 4.2. Spray Drift Measurements

**Figure 10.**
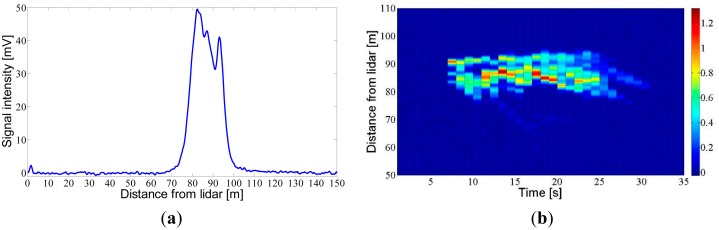
Detection of pesticide clouds generated by a cross-flow air-assisted sprayer. The sprayer was in a static position and located in a place with no crop. (**a**) Range profile of time-averaged lidar signal (100 laser pulses); (**b**) RTI plot of another pesticide cloud.

Figure 10 shows the measurements of two spray clouds generated by an air-assisted sprayer operating in an area without vegetation. These tests were conducted on 22 July 2014, in the ETSEA campus of the Universitat de Lleida, in Lleida (Catalonia, Spain). During these tests, the sprayer was kept in a static position at a distance of 90 m from the lidar system. Figure 10a shows the result of averaging 100 pulses (*PRF* = 2 Hz, observation time of 50 s) during which the sprayer was kept in continuous operation. The signal obtained shows two peaks which correspond to the two emission sides of the sprayer. Figure 10b shows a particular type of plot called *range-time intensity* (RTI). These were created from various consecutive measurements, in this case one each second, and show the evolution in time and distance of the cloud. The gradation of colours corresponds to the intensity of the received signal. In this test, spray measurement was performed from its initiation to its conclusion, with detection also of the residual drift in the air once the spraying had terminated.

**Figure 11.**
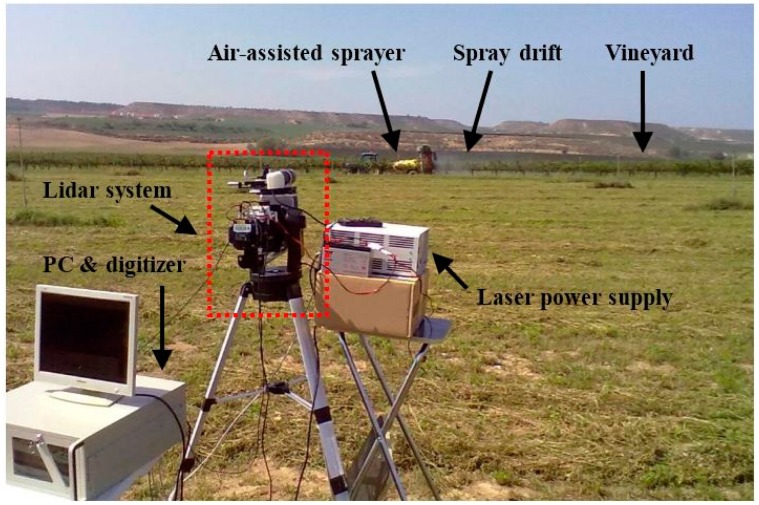
Lidar system deployed in the field during a spray drift measurement.

Figure 11 shows the typical set-up of a lidar system during a spray drift study. In this test, the spray drift was generated by an air-assisted sprayer treating a vineyard (background), while the lidar system is placed at a distance of 80 m away (foreground). The laser was pointed perpendicularly to the orchard and above it, to prevent any signal distortion by trees. The backscattered signal due to the interaction with the spray drift cloud is detected by the optoelectronic receiver and through the digitizer is sent to the PC so that the signal is displayed in real-time.

**Figure 12.**
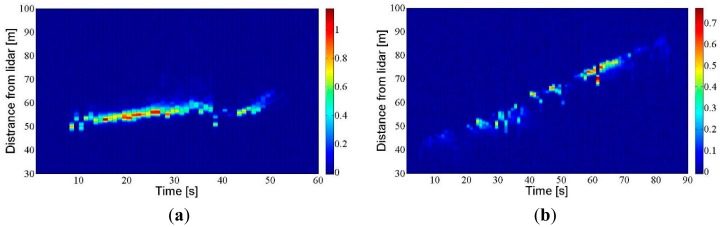
Detection of the pesticide spray drift in an apple orchard. (**a**) RTI plot corresponding to the sprayer in a static position; (**b**) RTI plot with the sprayer in movement with a constant speed.

The tests shown in Figure 12 were conducted on 24 July 2014, in an apple orchard attached to the ETSEA campus. The air-assisted sprayer was positioned in one of the orchard inter-rows while the laser beam was aimed at the neighbouring inter-row, parallel to it. The aim of the tests was to detect the fraction of spray able to get past the vegetation or, in other words, the spray drift. The drift generated during the application is shown in Figure 12a until the cloud signal dies out. In this test, the sprayer was kept in a static position so that the small variations in distance correspond to movement of the cloud caused by air currents. Figure 12b is similar to Figure 12a except that the sprayer was moved along the inter-row at constant speed as would take place in a real application. It can be seen how the drift cloud moves away as the sprayer advances along the inter-row.

## 5. Conclusions

In this work, the key parameters (wavelength, pulse energy, emission frequency, reception area, *etc.*) were determined for the design of an eye-safe lidar system for pesticide spray drift measurement. The methodology used is based on SNR simulations and on the study of the MPE for different wavelengths (905 nm, 1064 nm and 1.5 μm). A wavelength of 1.5 μm was chosen which, though requiring system constants between 2 and 3.6 times higher than at 905 nm, allows eye safety to be attained with reasonable expansion diameters, between one and two orders of magnitude below those at 905 or 1064 nm.

An erbium-glass laser based lidar prototype with 3-mJ of pulse energy, emitting at 1534 nm has been constructed. Initial tests with topographic targets and spray drift clouds have validated the correct operation of this instrument. The instrument that has been developed meets the design specifications that were established initially since it is capable of measuring mid-range spray drift as shown by the tests conducted, has high distance (2.4 m) and temporal (100 ms at maximum PRF) resolution, is eye-safe and weighs less than 15 kg (not including the data acquisition system). The cost of this prototype is about 40 k€, a value significantly lower than the price of lidar systems commonly used to atmospheric sounding. However, it should be noted that this prototype was developed in the framework of a research project and not as a commercial product.

A scanning system will need to be implemented in more advanced versions so that the system is able to provide a bi-dimensional image of pesticide plumes. Another aspect that will need to be examined is the possibility of using a coaxial configuration to reduce the minimum detection distance.

The availability of this instrument opens the door to the execution of a wide range of tests. The most immediate of these will comprise an intercomparison campaign with cooperative sensors capable of measuring the concentration and distribution of drift droplet sizes, in order to calibrate the lidar signal. It is expected that the developed instrument will enable estimation of the spray drift flux [μg/m^2^]. Flux measurement with the lidar may entail a substantial improvement over the present mass balance approach [36,37], the purpose of which is to quantify the fraction of the applied pesticide product which escapes from the treated area.

Consideration should also be given to the possible use of the developed instrument in other agroforestry applications such as measurement of the particulate matter (PM) generated in agricultural and livestock farming, the monitoring of sprinkler irrigation and fertilizer spraying or the prevention of forest fires. In relation with the latter application, previous studies [38,39] have proposed the development of a lidar system at the same wavelength (1.5 μm) as that used in the present work.

There is great potential in the use of lidar systems to monitor drift and agricultural air quality in general. The availability of the lidar system presented in this work and specifically designed for these applications, will enable better understanding of the phenomenon of drift and, as a result, the adoption of more efficient techniques to reduce or prevent its occurrence.

## Figures and Tables

**Table 1:** Opto-atmospheric parameters and solar background radiance for the studied wavelengths.

**λ**	**905 nm**	**1064 nm**	**1.5 μm**	**Spray Drift Cloud**
**α_tot_ [km^−1^]**	0.138	0.111	0.068	10
**β_tot_ [km^−1^·sr^−1^]**	5.813 × 10^−3^	4.883 × 10^−3^	3.306 × 10^−3^	0.5
**L_b_ [W·cm^−2^·nm^−1^·sr^−1^]**	10^−6^	4 × 10^−7^	4 × 10^−8^	-

**Table 2.** Required system constant, *K_s_*, for various combinations of design parameters at 905 nm.

**Variant Number**	**Photodetector**	**R_io_ [A/W]**	**M**	**F**	**NEP_m_ [** $\text{pW}/\sqrt{\text{Hz}}$**]**	**ξ_0_**	***K_b_* [m^2^·nm·sr]**	**Required *K_s_* [W·m^3^]**
**1**	Silicon APD	0.62	100	4	0.11	0.5	6.17 × 10^−9^	16.33
**2**	Silicon APD	0.62	100	4	0.11	0.25	6.17 × 10^−9^	32.65
**3**	Silicon APD	0.62	100	4	0.11	0.1	6.17 × 10^−9^	81.58
**4**	Silicon PIN	0.62	1	1	10.49	0.5	6.17 × 10^−9^	852.3
**5**	Silicon PIN	0.62	1	1	10.49	0.25	6.17 × 10^−9^	1705
**6**	Silicon PIN	0.62	1	1	10.49	0.1	6.17 × 10^−9^	4261

**Table 3.** Required system constant, *K_s_*, for various combinations of design parameters at 1064 nm.

**Variant Number**	**Photodetector**	**R_io_ [A/W]**	**M**	**F**	**NEP_m_ [** $\text{pW}/\sqrt{\text{Hz}}$**]**	**ξ_0_**	***K_b_* [m^2^·nm·sr]**	**Required *K_s_* [W·m^3^]**
**7**	Silicon APD	0.34	100	4	0.19	0.5	6.17 × 10^−9^	28.95
**8**	Silicon APD	0.34	100	4	0.19	0.25	6.17 × 10^−9^	57.88
**9**	Silicon APD	0.34	100	4	0.19	0.1	6.17 × 10^−9^	144.7
**10**	Silicon PIN	0.34	1	1	19.12	0.5	6.17 × 10^−9^	1513
**11**	Silicon PIN	0.34	1	1	19.12	0.25	6.17 × 10^−9^	3027
**12**	Silicon PIN	0.34	1	1	19.12	0.1	6.17 × 10^−9^	7568

**Table 4.** Required system constant, *K_s_*, for various combinations of design parameters at 1.5 μm.

**Variant Number**	**Photodetector**	**R_io_ [A/W]**	**M**	**F**	**NEP_m_ [** $\text{pW}/\sqrt{\text{Hz}}$**]**	**ξ_0_**	***K_b_* [m^2^·nm·sr]**	**Required *K_s_* [W·m^3^]**
**13**	InGaAs APD	0.93	10	5.5	0.7	0.5	6.17 × 10^−9^	58.29
**14**	InGaAs APD	0.93	10	5.5	0.7	0.25	6.17 × 10^−9^	116.6
**15**	InGaAs APD	0.93	10	5.5	0.7	0.1	6.17 × 10^−9^	291.5
**16**	InGaAs PIN	0.93	1	1	7	0.5	6.17 × 10^−9^	530.7
**17**	InGaAs PIN	0.93	1	1	7	0.25	6.17 × 10^−9^	1061
**18**	InGaAs PIN	0.93	1	1	7	0.1	6.17 × 10^−9^	2654

**Table 5.** Required beam diameters (in mm) at several repetition rates for the studied wavelengths and pulse energies.

**Wavelength**	**905 nm**			**1064 nm**			**1.5 μm**		
Pulse Energy	15 μJ	75 μJ	300 μJ	25 μJ	100 μJ	500 μJ	50 μJ	200 μJ	1 mJ
1 Hz	51	115	230	34	67	151	0.8	1.6	3.6
10 Hz	69	153	307	45	90	201	2.5	5.0	11
100 Hz	91	204	409	60	120	268	8.0	16	36

**Table 6.** System specifications.

**Emitter**	Laser	Model	Kigre™ MK-85
		Centre wavelength, λ	1534 nm (Erbium glass laser)
		Spectral bandwidth	4.5 nm (FWHM)
		Pulse energy, *E*_0_	3 mJ
		Pulse duration, τ*_l_*	6 ns
		Pulse repetition frequency, *PRF*	Single shot—10 Hz (adjustable)

	Beam Expander	Output beam diameter	16 mm
		Beam expansion	20×
		Output beam divergence, θ	∼210 μrad (full angle)

**Receiver**	Telescope	Model	Meade™ ETX80
		Primary lens diameter, *d*_0_	80 mm

	Interference Filter	Centre wavelength, λ	1530 nm
		Full width at half maximum, Δλ	12 nm

	APD Module	Model	Thorlabs™ APD110C
		Active area diameter, *d_D_*	0.2 mm
		Intrinsic Responsivity, *R_io_*	0.9 A/W (1500 nm)
		Noise Equivalent Power, *NEP_m_*	0.46 pW/Hz^1/2^
		APD gain, *M*	10
		Transimpedance gain, *G*	10^5^ V/A
		Output bandwidth (3 dB), *B_N_*	DC—50 MHz

	Digitizer	Model	GaGe™ CompuScope 12502
		Sampling rate	500 MS/s
		Resolution	12 bits

## References

[1] Gregorio E., Rosell-Polo J.R., Sanz R., Rocadenbosch F., Solanelles F., Garcerá C., Chueca P., Arnó J., del Moral I., Masip J. (2014). LIDAR as an alternative to passive collectors to measure pesticide spray drift. Atmos. Environ..

[2] Hoff R.M., Mickle R.E., Froude F.A. (1989). A rapid acquisition lidar for aerial spray diagnostics. Trans. ASAE.

[3] Mickle R.E. (1994). Utilizing vortex behaviour to minimize drift. J. Environ. Sci. Health Part B.

[4] Mickle R.E. (1996). Influence of aircraft vortices on spray cloud behaviour. J. Am. Mosq. Control Assoc..

[5] Stoughton T.E., Miller D.R., Yang Y., Ducharme K.M. (1997). A comparison of spray drift predictions to lidar data. Agric. For. Meteorol..

[6] Miller D.R., Stoughton T.E. (2000). Response of spray drift from aerial applications at forest edge to atmospheric stability. Agric. For. Meteorol..

[7] Miller D., Salyani M., Hiscox A. Remote measurement of spray drift from orchard sprayer using LIDAR.

[8] Hiscox A.L., Miller D.R., Nappo C.J., Ross J. (2006). Dispersion of fine spray from aerial applications in stable atmospheric conditions. Trans. ASABE.

[9] Khot L.R., Miller D.R., Hiscox A.L., Salyani M., Walker T.W., Farooq M. (2011). Extrapolation of droplet catch measurements in aerosol application treatments. At. Sprays.

[10] Tsai M.Y. (2007). The Washington Spray Drift Studies: Understanding the Broader Mechanisms of Pesticide Spray Drift. Ph.D Thesis.

[11] Gregorio E., Solanelles F., Rocadenbosch F., Rosell J.R., Sanz R. (2011). Airborne spray drift measurement using passive collectors and lidar systems. Proc. SPIE.

[12] Gimmestad G.G., Roberts D.W., Stewart J.M., West L.L., Wood J.W. NEXLASER—An unattended tropospheric ozone and aerosol lidar—First results.

[13] Spinhirne J.D. (1993). Micro-pulse Lidar. IEEE Trans. Geosci. Remote Sens..

[14] Gregorio E., Rocadenbosch F., Tiana-Alsina J., Comerón A., Sanz R., Rosell J.R. (2012). Parameter design of a biaxial lidar ceilometer. J. Appl. Remote Sens..

[15] Rocadenbosch F., Soriano C., Comerón A., Baldasano J.M., Rodríguez A., Muñoz C., García-Vizcaino D. (2001). 3D scanning portable backscatter lidar platform for atmospheric remote sensing: Performance and architecture overview. Proc. SPIE.

[16] Mayor S.D., Spuler S.M., Morley B.M., Loew E. (2004). Raman-shifted eye-safe aerosol lidar. Appl. Opt..

[17] Gimmestad G.G., Roberts D.W. 1.5 Microns: The future of unattended aerosol lidar?.

[18] Collis R.T.H., Russell P.B., Hinkley E.D. (1976). Lidar measurement of particles and gases by elastic backscattering and differential absorption. Laser Monitoring of the Atmosphere.

[19] Koschmider H. (1924). Theorie der horisontalen Sichtweite. Beitr. Phys. Freien Atmos..

[20] Measures R. (1992). Laser Remote Sensing: Fundamentals and Applications.

[21] Morille Y., Haeffelin M., Drobinski P., Pelon J. (2007). STRAT: An automated algorithm to retrieve the vertical structure of the atmosphere from single-channel lidar data. J. Atmos. Ocean. Technol..

[22] Carlson A.B. (1986). Communication Systems: An Introduction to Signals and Noise in Electrical Communication.

[23] Kovalev V.A., Eichinger W.E. (2004). Elastic Lidar: Theory, Practice, and Analysis Methods.

[24] Dunai D., Zoletnik S., Sarkozi J., Field R. (2010). Avalanche photodiode based detector for beam emission spectroscopy. Rev. Sci. Instrum..

[25] Spuler S.M., Mayor S.D. (2005). Scanning eye-safe elastic backscatter lidar at 1.54 μm. J. Atmos. Ocean. Technol..

[26] Patterson E.M., Roberts D.W., Gimmestad G.G. (1989). Initial measurements using a 1.54-μm eyesafe Raman shifted lidar. Appl. Opt..

[27] Carnuth W., Trickl T. (1994). A powerful eyesafe infrared aerosol lidar: Application of stimulated Raman backscattering of 1.06 micron radiation. Rev. Sci. Instrum..

[28] Spinhirne J.D., Chudamani S., Cavanaugh J.F., Bufton J.L. (1997). Aerosol and cloud backscatter at 1.06, 1.54, and 0.53 μm by airborne hard-target-calibrated Nd:YAG/methane Raman lidar. Appl. Opt..

[29] Mayor S.D., Spuler S.M. (2007). Polarization lidar at 1.54 μm and observations of plumes from aerosol generators. Opt. Eng..

[30] Hecht J. (2008). Understanding Lasers: An Entry-Level Guide.

[31] Harrell S.R., Wilcox W., Killinger D., Rines G.A., Schwarz R. (1995). High power, eye-safe 1.57 micron OPO lidar for atmospheric boundary layer measurements. Proc. SPIE.

[32] Gong W., Chyba T.H., Temple D.A. (2007). Eye-safe compact scanning LIDAR technology. Opt. Lasers Eng..

[33] Setzler S.D., Francis M.P., Young Y.E., Konves J.R., Chicklis E.P. (2005). Resonantly pumped eyesafe erbium lasers. IEEE J. Sel. Top. Quantum Electron..

[34] Gaumet J.L., Heinrich J.C., Cluzeau M. (1998). Cloud-base height measurements with a single-pulse erbium-glass laser ceilometer. J. Atmos. Ocen. Technol..

[35] Lavrov A., Utkin A.B., Vilar R. (2010). Simple eye-safe lidar for cloud height measurement and small forest fire detection. Opt. Spectrosc..

[36] Balsari P., Marucco P., Tamagnone M. (2004). A system to assess the mass balance of spray applied to tree corps. Trans. ASAE.

[37] Salyani M., Farooq M., Sweeb R.D. (2007). Spray deposition and mass balance in citrus orchard applications. Trans. ASABE.

[38] Vilar R., Lavrov A. (2000). Estimation of required parameters for detection of small smoke plumes by lidar at 1.54 μm. Appl. Phys. B.

[39] Utkin A.B., Lavror A.V., Costa L., Simoes F., Vilar R. (2002). Detection of small forest fires by lidar. Appl. Phys. B.

